# Meta-Analyses of *KIF6* Trp719Arg in Coronary Heart Disease and Statin Therapeutic Effect

**DOI:** 10.1371/journal.pone.0050126

**Published:** 2012-12-07

**Authors:** Ping Peng, Jiangfang Lian, R. Stephanie Huang, Limin Xu, Yi Huang, Yanna Ba, Xi Yang, Xiaoyan Huang, Changzhen Dong, Lina Zhang, Meng Ye, Jianqing Zhou, Shiwei Duan

**Affiliations:** 1 Ningbo Medical Center, Lihuili Hospital, Ningbo University, Ningbo, Zhejiang, China; 2 The Affiliated Hospital, School of Medicine, Ningbo University, Ningbo, Zhejiang, China; 3 Department of Medicine, University of Chicago, Chicago, Illinois, United States of America; University of Milan, Italy

## Abstract

**Aims:**

The goal of our study is to assess the contribution of *KIF6* Trp719Arg to both the risk of CHD and the efficacy of statin therapy in CHD patients.

**Methods and Results:**

Meta-analysis of 8 prospective studies among 77,400 Caucasians provides evidence that 719Arg increases the risk of CHD (*P*<0.001, HR = 1.27, 95% CI = 1.15–1.41). However, another meta-analysis of 7 case-control studies among 65,200 individuals fails to find a significant relationship between Trp719Arg and the risk of CHD (*P* = 0.642, OR = 1.02, 95% CI = 0.95–1.08). This suggests that the contribution of Trp719Arg to CHD varies in different ethnic groups. Additional meta-analysis also shows that statin therapy only benefit the vascular patients carry 719Arg allele (*P*<0.001, relative ratio (RR) = 0.60, 95% CI = 0.54–0.67). To examine the role of this genetic variant in CHD risk in Han Chinese, we have conducted a case-control study with 289 CHD cases, 193 non-CHD controls, and 329 unrelated healthy volunteers as healthy controls. On post hoc analysis, significant allele frequency difference of 719Arg is observed between female CHD cases and female total controls under the dominant model (*P* = 0.04, χ^2^ = 4.228, df = 1, odd ratio (OR) = 1.979, 95% confidence interval (CI) = 1.023–3.828). Similar trends are observed for post hoc analysis between female CHD cases and female healthy controls (dominant model: *P* = 0.04, χ^2^ = 4.231, df = 1, OR = 2.015, 95% CI = 1.024–3.964). Non-genetic CHD risk factors are not controlled in these analyses.

**Conclusions:**

Our meta-analysis demonstrates the role of Trp719Arg of *KIF6* gene in the risk of CHD in Caucasians. The meta-analysis also suggests the role of this variant in statin therapeutic response in vascular diseases. Our case-control study suggests that Trp719Arg of *KIF6* gene is associated with CHD in female Han Chinese through a post hoc analysis.

## Introduction

Severe coronary artery disease (CAD), also called coronary heart disease (CHD), is characterized by occlusive epicardial coronary artery stenosis. CHD complications such as myocardial infarction (MI) are the leading causes of death in the United States [1] and worldwide, with over 500,000 and 7,000,000 deaths per year in the United States and worldwide, respectively [2]. The most generally accepted hypothesis is that CHD is a complex disease, resulting from the interaction of multiple genes and together with environmental factors [3]. Current genome-wide association studies (GWAS) have identified a handful of genetic variants underlying the risk of CHD. However, over 95% of the genetic variants in disease risk remains unknown and warrant further investigation [4], [5].

Kinesin like protein 6 (*KIF6*) gene encodes an intracellular motor protein transporting cellular cargos along microtubules in an ATP dependent process. *KIF6* gene is expressed in many tissues and cell types including coronary arteries and vascular cells [6], [7], [8], [9]. Single nucleotide polymorphisms (SNPs) of *KIF6* such as Trp719Arg (rs20455) have been shown to be associated with the risk of CHD [10], [11], [12]. Genome-wide association studies (GWAS) have demonstrated that 719Arg can increase the risk of CHD in Europeans and North Americans [6], [10], [11], [12], [13], [14], [15], [16]. Moreover, 719Arg carriers may have better statin therapeutic effects that includes the effect of reducing the low-density lipoprotein cholesterol (LDL-C) levels, and other pleiotropic effects on inflammation, thrombogenesis, and arterial vasomotor function [8], [12], [17].

Despite this evidence, no significant association has been observed in different populations [6], [9], [16], [18], [19]. These discrepancies suggest that the role of Trp719Arg in the risk of CHD may vary for different ethnic groups. The goals of our study are to summarize the contribution of Trp719Arg to the risk of CHD and the therapeutic effect of statins both in the meta-analysis fashion with various ethnic groups as well as a focused study in Han Chinese.

## Materials and Methods

### Retrieval of published studies

To perform meta-analysis, we systematically search for available articles in English or Chinese from 2005 to 2011 in multiple electronic databases, including PubMed, EMbase, China National Knowledge Infrastructure (CNKI), Wanfang Chinese Periodical Database and Web of Science. The search keywords apply the MeSH (Medical Subject Headings in the US National Library of Medicine) terms that include “coronary heart disease” or “coronary artery disease” or “myocardial infarction” combined with “KIF6” or “kinesin like protein 6” or “rs20455” or “719Arg”, “polymorphism”, “genetic association” and/or “statin” [20]. We read the full text articles to collect the relevant information. The related articles in the MEDLINE option as well as reference lists of all retrieved studies are also checked for citations of other relevant publications that are not identified initially [21]. The included studies have to satisfy the following criteria: 1) they have been published as articles or letters in peer-reviewed journals, 2) had a case-control design or a nested case-control design within a prospective study and reported their results by genotype, or had sufficient published data on ORs or HRs and 95% CIs, or genotype and allele frequencies to determine an measure of relative risk [22].

### Study selection

Data extraction is carried out by at least two reviewers (PP and LMX) on a standard protocol, and the consensus data are established by discussion. In the meta-analyses, the following data collection is included: name of the first author, publication year, country, ethnic population, study stage, numbers of individual in the case and the control groups and prospective studies, OR, RR, HR and 95% CI. The meta-analyses are performed by Stata software (version 11.0, Stata Corporation, College Station, TX) [23]. Publication bias is visualized by funnel plots and Egger regression plot [24].

### Han Chinese case-control study sample collection

A total of 289 CHD patients and 193 non-CHD patients are collected between May of 2008 and November of 2011 from the Lihuili Hospital in Ningbo city of Zhejiang province, China. Patients are differentiated into case and control group by standardized coronary angiography according to the Seldinger's method [25]. Each patient is judged by at least two independent cardiologists. The inclusion of CHD cases requires the evidence of the coronary artery stenosis greater than or equal to 50% occlusion of one or more major coronary arteries [26], [27] or a history of prior angioplasty or coronary artery bypass surgery. Non-CHD patients have a less than 50% occlusion in any major coronary artery, and do not have any atherosclerotic vascular disease. In addition, 329 healthy persons originated from Ningbo city are recruited as healthy controls who are excluded from any congenital heart disease, cardiomyopathy, liver or renal disease. All subjects are Han Chinese originated from Ningbo city in the Eastern China. The study protocol has been approved by the Ethical Committee of Lihuili Hospital in Ningbo, and the informed written consent has been obtained from all subjects. Blood samples are collected in 3.2% citrate sodium-treated tubes and then stored at −80°C. All blood samples of cases and controls are collected by the same investigators.

### SNP genotyping

Human genomic DNA is isolated from peripheral blood using a conventional phenol/chloroform extraction method, and is quantified using the PicoGreen® double strand (dsDNA) DNA Quantification Kit (Molecular Probes, Inc. Eugene, USA). Amplification is performed on the ABI Geneamp® PCR System 9700 Dual 384-Well Sample Block Module (Applied Biosystems, Foster City, CA) for the Polymerase Chain Reaction (PCR). The sense primer is 5′-ACGTTGGATGTTCTCCAGACATCTGACTCC-3′ and the antisense primer is 5′-ACGTTGGATGCCGGTGAGTTCTCACCTTAC-3′. PCR conditions include an initial denaturation stage 94°C for 15 sec, followed by 45 cycles at 94°C for 20 sec, 56°C for 30 sec, primer extension at 72°C for 1 min, and a final extension for 3 minutes at 72°C. Primer Extension for genotyping is performed on the SEQUENOM® Mass-ARRAY iPLEX® platform according to the manufacturer's instructions [28].

### Statistical analyses

Genotype distribution of different sample groups is tested for departure from Hardy-Weinberg equilibrium (HWE) by the Arlequin program (version 3.5) [29], [30]. We use the SPSS statistical software (version 18) to compare the differences in the genotype and allele frequencies between CHD cases and two controls (including diagnosed controls and healthy controls) [31]. OR values of genotypes are determined by comparing the heterozygous or homozygous genotype to wild type. The 95% CI are calculated using the SPSS statistical software (version 18) [27]. The power of the study is determined by Power and Sample Size Calculation software (v3.0.43) [32]. A two-tailed value of *P*<0.05 is considered to be significant.

## Results

**Figure 1:**
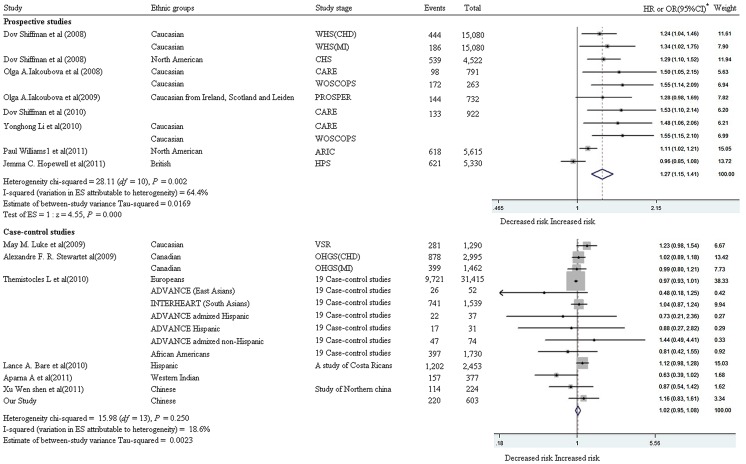
Meta-analyses between Trp719Arg and CHD. a: CARE (the Cholesterol and Recurrent Events study); WOSCOPS (the West of Scotland Coronary Prevention Study); PROSPER (PROspective Study of Pravastatin in the Elderly at Risk); HPS (the Heart Protection Study); ARIC (the Atherosclerosis Risk in Communities study); CHS (the Cardiovascular Health Study); WHS (the Woman's Health Study); OHGS (Ottawa Heart Genomics Study); VSR (Vienna Stroke Registry); *: Reference allele is 719Arg.

**Figure 2:**
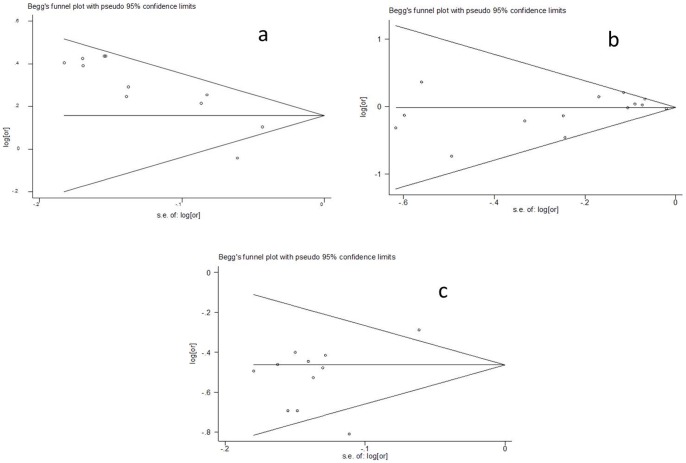
Funnel plots for association studies between Trp719Arg and CHD. a: Meta-analysis of 8 perspective studies; b: Meta-analysis of 7 case-control studies; c: Meta-analysis of 8 studies between KIF6 Trp719Arg and statin response.

A total of 14 published articles [6], [8], [9], [10], [11], [13], [14], [15], [18], [19], [29], [33], [34], [35] are eligible for the meta-analysis of Trp719Arg to the risk of CHD. Among these studies, 8 are prospective studies with 77,400 individuals who are Caucasians in Europe and North America; while the other 6 studies are case-control studies with 64,400 individuals from various ethnic groups, comprising Europeans or European descendents, Hispanics, African American and Asians. As shown in Figure 1, we have found a significant contribution of 719Arg allele to the risk of CHD (*P*<0.001, the overall HR = 1.27, 95% CI = 1.15–1.41) and a high heterogeneity among the 8 prospective studies (I^2^ = 64.4%, *P* = 0.002, χ^2^ = 28.11, df = 10). In contrast, a low heterogeneity is observed in the meta-analysis of the six case-control studies and our study (I^2^ = 18.6%, χ^2^ = 15.98, df = 13, *P* = 0.25). The latter meta-analysis has not found a significant association between 719Arg and the risk of CHD (*P* = 0.642, the overall OR = 1.02, 95% CI = 0.95–1.08) despite of the large sample size (65,200 individuals). This observation along with the mixed ethnicity in this meta-analysis, we speculates that the contribution of Trp719Arg to the risk of CHD varies in different ethnic groups. No publication bias is observed among the involved studies in the two meta-analyses (Figure 2a and 2b).

**Figure 3:**
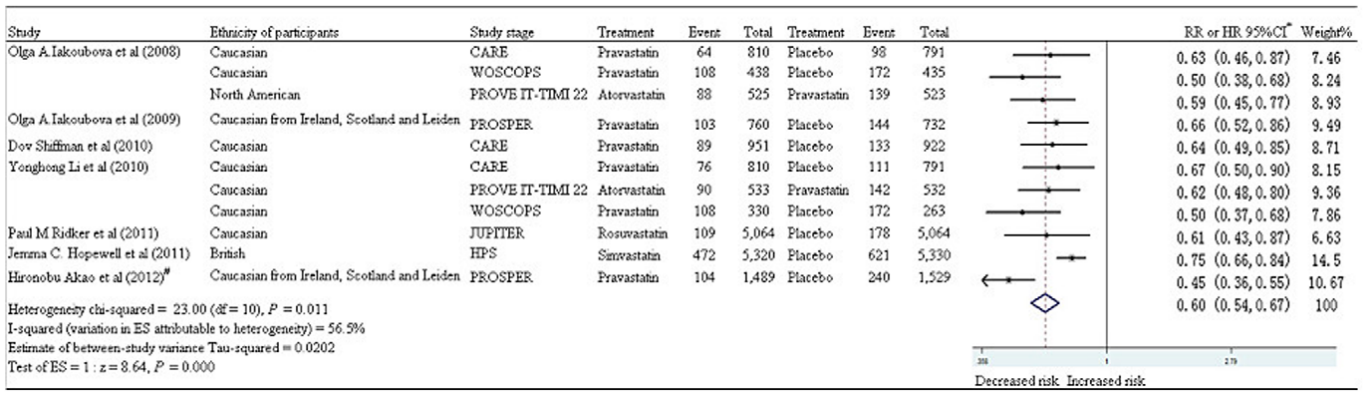
Meta-analysis between Trp719Arg and statin therapy. *: Reference allele is 719Arg; Weights are from random effects analysis; #: The study's RR is calculated using the SPSS statistical software (version 18).

To assess the role this genetic variant in statin response, a meta-analysis has also been performed using data from eight association studies [8], [10], [13], [15], [16], [17], [36], [37]. As shown in Figure 3, a significant reduction of the number of deaths or major cardiovascular events in the 719Arg carriers is observed (P<0.001, overall RR = 0.60, 95% CI = 0.54–0.67). High heterogeneity (I^2^ = 56.5%, *P* = 0.011, χ^2^ = 23, df = 10) is found among the eight studies. Random effects analysis model is used for the meta-analysis. No publication bias is observed among the involved studies by the funnel plot (Figure 2c).

To test the specific role of Trp719Arg in CHD risk in Han Chinese, we have conducted a case-control study focusing on this ethnic group. Genotypic and allelic comparison of *KIF6* Trp719Arg between CHD cases and different controls are shown in Table 1. No departure of HWE is observed for Trp719Arg. The 719Arg allele frequencies are 0.503 in CHD cases, 0.482 in non-CHD controls, and 0.483 in healthy controls and 0.483 in total controls. These are similar to the allele frequency reported by HapMap in Asian populations (0.570 in HapMap-CHB and 0.477 in HapMap-JPT). No significant differences are observed in the genotype and allele distribution between CHD cases and each of three controls (Table 1) regardless the genetic model evaluated (Table S1 and S2). Furthermore, when we stratify the data analysis into each sex group with respect to allele and genotype frequencies, we haven't found any significant association of *KIF6* Trp719Arg with the risk of CHD either (Table 2). However, on post hoc analysis we observe a departure from HWE in female CHD cases with an excess of heterozygotes (*P* = 0.041). Interestingly, an association test in females shows significant different distribution of 719Arg-containing genotypes between CHD cases and healthy controls under the dominant model (Table 3, *P* = 0.04, χ^2^ = 4.231, df = 1, OR = 2.015, 95% CI = 1.024–3.964). This finding remains true between CHD cases and total controls under the dominant model for females (P = 0.04, χ^2^ = 4.228, df = 1, OR = 1.979, 95% CI = 1.023–3.828). Note that post hoc analysis of our results generates the hypothesis that 719Arg tends to have a dominant effect on the risk of CHD (OR = 2.012) only in female Eastern Han Chinese (Table 3 and Table S3).

**Table 1:** Genotype and allele of Trp719Arg in case and control groups.

Group(rs20455)	Genotype			?^2^	*P(d.f. = 2)*	Allele		?^2^	*P(d.f. = 1)*	OR(95%CI)	HWE
	719Arg/719Arg	719Arg/719Trp	719Trp/719Trp			719Arg	719Trp				
CHD cases	71	149	69			291	287				0.642
non-CHD controls	47	92	54	1.116	0.570	186	200	0.432	0.511	1.090(0.843–1.411)	0.563
Healthy controls	74	170	85	0.520	0.786	318	340	0.501	0.479	1.084(0.867–1.356	0.584
Total controls	121	262	139	0.769	0.679	504	540	0.638	0.424	1.086(0.887–1.331)	0.931

**Table 2:** Genotype and allele of Trp719Arg in male and female subgroups.

Group(rs20455)	Total	Genotype			?^2^	*P(d.f. = 2)*	Allele		?^2^	*P(d.f. = 1)*	0R	95%CI	HWE
		719Arg/719Arg	719Arg/719Trp	719Trp/719Trp			719Arg	719Trp					
Male													
CHD cases	208	53	98	57			204	212					0.409
non-CHD controls	97	21	46	30	0.690	0.708	88	106	0.717	0.397	1.159	0.824–1.631	0.684
Healthy controls	85	17	47	21	1.739	0.419	81	89	0.094	0.760	1.057	0.740–1.511	0.387
Total controls	182	38	93	51	1.209	0.546	169	195	0.530	0.467	1.11O	0.838–1.472	0.769
Female													
CHD cases	80	18	50	12			86	74					0.041
non-CHD controls	96	26	46	24	4.201	0.122	98	94	0.257	0.612	1.115	0.732–1.697	0.689
Healthy controls	244	57	123	64	4.907	0.086	237	251	1.295	0.255	1.231	0.861–1.760	1.000
Total controls	340	83	169	88	5.353	0.069	335	345	1.042	0.307	1.197	0.847–1.690	0.914

**Table 3:** The association of Trp719Arg with gender risk in the dominant model.

rs20455	Total	Genotype		?^2^	*P(d.f. = 1)*	OR	95%CI
		719Arg/719Arg+719Arg/719Trp	719Trp/719Trp				
Male							
CHD cases	208	151	57				
non-CHD controls	97	67	30	0.403	0.526	1.186	0.700–2.010
Healthy controls	85	64	21	0.225	0.635	0.869	0.487–1.552
Total controls	182	131	51	0.019	0.892	1.031	0.661–1.608
Female							
CHD cases	80	68	12				
non-CHD controls	96	72	24	2.682	0.101	1.889	0.876–4.072
Healthy controls	244	180	64	4.231	**0.040**	2.015	1.024–3.964
Total controls	340	252	88	4.228	**0.040**	1.979	1.023–3.828

A power calculation shows that our study only has a 20.6% power to detect a relative risk of 719Arg at a significant level of 0.05, suggesting that a lack of power is likely to explain our failure to find a significant association (Table 1 and 2).

## Discussion

Several lines of evidence have shown that 719Arg is likely to increase the risk of CHD [14]. In the Cardiovascular Health Study (CHS), a population-based investigation of 3,849 white Americans has found that 719Arg is associated with the risk of cardiovascular disease [14]. Two prospective trials comprising the Cholesterol and Recurrent Events (CARE) and the West of Scotland Coronary Prevention Study (WOSCOPS) have revealed 719Arg as a CHD risk factor among a total of over 4,000 Caucasian participants [10]. Another investigation among 25,283 initially healthy Caucasian women, namely Women's Health Study (WHS), has found that females with 719Arg allele of *KIF6* have 34% higher risk of AMI and 24% higher risk of CHD [6]. Under a dominant model, our post hoc analysis reveals the contribution of 719Arg to the higher risk of CHD in females (*P* = 0.04, χ^2^ = 4.231, df = 1, OR = 2.015, 95% CI = 1.024–3.964). This female-specific finding agrees with the observations in a total of 25,283 Caucasian women enrolled in the WHS [6]. Our meta-analyses among 77,400 Caucasians provides evidence that 719Arg increases the risk of CHD (*P*<0.001, HR = 1.27, 95% CI = 1.15–1.41). This result agrees with a previous meta-analysis that has found a 20% increase in the risk of CHD for the 719Arg carriers [12].

We also notice that there is an ethnic difference in the frequency of 719Arg allele. In our healthy controls, it is 0.483 that is similar to 0.570 in HapMap-CHB, 0.477 in HapMap-JPT, and 0.51 in the Indian population [33]. However, much lower frequency of 719Arg allele is observed in Europeans (0.358 in HapMap-CEU) and Japanese (0.386) [33]. Interestingly, the latter is much lower than 0.477 in HapMap-JPT that consists of 90 Japanese individuals. The Costa-Rican population, an admixture of three populations, Southern Europeans, Amerindians, and West Africans, has a minor allele frequency of 0.345 [9]. It is interesting that the 719Arg allele frequency is very high in the Sub-Saharan African population (0.908) [33]. These ethnic differences imply that further replication of 719Arg to the risk of CHD in other populations is warranted.

No significant association is found between 719Arg and the risk of CHD in the meta-analysis of 7 case-control studies among 65,200 individuals. The recruited participants in the meta-analysis are from several different ethnic populations including Europeans or European descendents, African descendents in America, East Asians, and South Asians. Among these case-control studies, a large one with a total of 17,000 cases and 39,369 controls failed to replicate the association between Trp719Arg and the risk of clinical CHD in multiple ethnic populations [34]. The contribution of 719Arg to the risk of CHD was unable to be replicated in the Costa Rican [9] and the Western Indian [33]. The conflicting results may be explained by the survival bias and drug interaction that can attenuate the case-control comparisons of Trp719Arg [11], or it could be also due to the lack of genetic effect in certain ethnic groups.

An allele-specific model of 719Arg is observed in the statin therapy of coronary events. Significantly reduced coronary events and other major vascular events have been observed in 719Arg carriers but not in non-carriers [10], [12], [13], although a large primary prevention trial JUPITER study with 8,781 Caucasian trial participants has found no difference in the rosuvastatin therapeutic outcomes between carriers (*P* = 0.007, HR = 0.61, 95% CI = 0.43–0.87) and non-carriers (*P* = 0.009, HR = 0.59, 95% CI = 0.39–0.88) of 719Arg [17]. Our meta-analysis has found that statin therapy receives significant benefit only in the carriers of 719Arg (P<0.001, overall RR = 0.60, 95% CI = 0.54–0.67).

Mechanistically, the structure of KIF6 protein consists of a conserved motor domain and a non-conserved tail domain. The conserved motor domain can propel the kinesin along microtubules in an ATP-dependent manner. The non-conserved tail domain binds to its cargoes such as membrane organelles, protein complexes, and mRNAs [10]. Trp719Arg is located in a predicted coiled-coil structure of the non-conserved tail domain. This variant causes a basic arginine residue to be replaced with a nonpolar tryptophan residue, and thus it might affect the cargo binding of the kinesin [37]. The higher risk estimates for heterozygotes could indicate that there is a functional difference between heterodimers and homodimers of the KIF6 protein, possibly because the Arg-Trp heterodimers differ from Arg-Arg and Trp-Trp homodimers in their stability or in their ability to transport cargo [16]. KIF6 may play a role in cell shape remodeling, however, the pathophysiologic role of Trp719Arg of *KIF6* gene in CHD risk and coronary event reduction from statin therapy has yet to be clearly elucidated [12], [14].

There are several limitations in our case-control study focused on Han Chinese. Firstly, sample size in our study is comparatively small and it has only 20.6% power to detect the association of Trp719Arg with CHD at a significant level of 0.05. In addition, only non-fatal CHD cases are recruited in the present study. The 719Arg allele is hypothesized to increase the risk of incident fatal CHD more than the risk of incident nonfatal CHD [34], the exclusion of fatal CHD cases might attenuate the detection of a significant association between the SNP and CHD. Secondly, the genotype distribution of Trp719Arg in female CHD patients has an excess of heterozygotes (HWE test: *P* = 0.041). This phenomenon may be due to the improperly pair-wised design, the small population size or that the female CHD patients do not obey HWE. Therefore, we need to take caution with the significant association results in females under the dominant model. Thirdly, the information of statin usage is not available in our samples. Our study might underestimate the risk of this genetic variation for a potential bias by the statin therapy. Moreover, CHD is strongly correlated with smoking status, LDL-C/HDL-C, history of hypertension or diabetes, and body mass index. Our genetic testing has not been adjusted with those risk factors. Fourthly, gender has been shown to be an important modifier of cardiovascular disease risk [38]. Our previous study [39] has found a female-dependent association between a *PDGFD* gene variation (rs974819) and CHD risk. In the present meta-analyses, we are unable to make a gender adjustment or stratification for all the involved studies. Finally, our study isn't designed to test whether *KIF6* variants are associated with the statin therapy outcome.

In conclusion, our meta-analyses over 143,000 individuals have shown that 719Arg is a risk factor of CHD in Caucasians but its effects on CHD may vary in other ethnic populations. Meta-analysis also indicated that statin therapy may selectively benefit patients with *KIF6* 719Arg allele. Despite failing to find a significant relationship between Trp719Arg and CHD in Eastern Han Chinese for the study population as a whole, post hoc analysis reveals a female-specific association. This gender-specific finding should be investigated future studies.

## Supporting Information

Table S1 Association of Trp719Arg with CHD in the dominant model.
(DOCX)

Table S2 Association of Trp719Arg with CHD in the recessive model.
(DOCX)

Table S3 Association of Trp719Arg with gender risk in the recessive model.
(DOCX)

Flow Diagram S1 **PRISMA flow diagram.**
(DOC)

Checklist S1 **PRISMA checklist.**
(DOC)
