# Supplementary material for: Meta-Analyses of KIF6 Trp719Arg in Coronary Heart Disease and Statin Therapeutic Effect
Source: PLoS One. 2012 Dec 7;7(12):e50126. doi: 10.1371/journal.pone.0050126 (PMC3517591; doi:10.1371/journal.pone.0050126)
Supplement: Table S2 — Association of Trp719Arg with CHD in the recessive model. (DOCX) [file pone.0050126.s002.docx]

**Table S2: Association of Trp719Arg with CHD in the recessive model**
