# Supplementary material for: Meta-Analyses of KIF6 Trp719Arg in Coronary Heart Disease and Statin Therapeutic Effect
Source: PLoS One. 2012 Dec 7;7(12):e50126. doi: 10.1371/journal.pone.0050126 (PMC3517591; doi:10.1371/journal.pone.0050126)
Supplement: Flow Diagram S1 — PRISMA flow diagram. (DOC) [file pone.0050126.s004.doc]

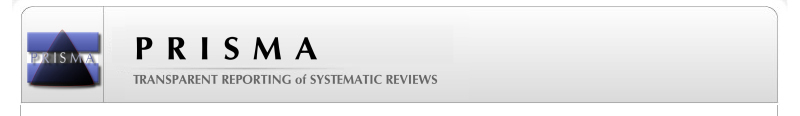
**PRISMA 2009 Flow Diagram**

**Screening**

**Included**

**Eligibility**

**Identification**

Records identified through database searching of PubMed, Embase, and Web of Science from 2005 to 2011
(n = 37)

Additional Chinese records identified through other sources of CNKI and Wanfang from 2005 to 2011
(n = 1)

Records after duplicates removed
(n =38 )

Records screened
(n = 38 )

Records excluded
(n =0 )

Full-text articles assessed for eligibility
(n =38 )

Full-text articles excluded, with reasons
(n = 0 )

Studies included in qualitative synthesis
(n =38 )

Studies included in quantitative synthesis (meta-analysis, n =19)

Meta-analyses of KIF6 Trp719Arg in coronary heart disease

(n =14)^[ 6, 8, 9, 10, 11, 13, 16, 18, 19, 32, 33, 34, 35,36]^

Meta-analyses of KIF6 Trp719Arg in statin therapeutic effect

(n =8)^[ 8, 10, 14, 15, 16, 17, 37, 38]^
