# Supplementary material for: Meta-Analyses of KIF6 Trp719Arg in Coronary Heart Disease and Statin Therapeutic Effect
Source: PLoS One. 2012 Dec 7;7(12):e50126. doi: 10.1371/journal.pone.0050126 (PMC3517591; doi:10.1371/journal.pone.0050126)
Supplement: Checklist S1 — PRISMA checklist. (DOC) [file pone.0050126.s005.doc]

| **Section/topic** | **#** | **Checklist item** | **Reported on page #** |
| --- | --- | --- | --- |
| **TITLE** | | |  |
| Title | 1 | A case-control study and meta-analyses of KIF6 Trp719Arg in coronary heart disease and statin therapeutic effect | 1 |
| **ABSTRACT** | | |  |
| Structured summary | 2 | Aims: The goal of our study is to access the contribution of KIF6 Trp719Arg to both the risk of CHD and the efficacy of statin therapy in CHD patients.  Methods and results: A total of 289 CHD cases and 193 non-CHD controls are recruited from Lihuili Hospital in Ningbo city. Another 329 unrelated healthy volunteers are selected as healthy controls. Significant difference of 719Arg allele frequency is observed between female CHD cases and female total controls under the dominant model (P = 0.04, χ2 = 4.228, df = 1, odd ratio (OR) = 1.979, 95% confidence interval (CI) = 1.023-3.828). Similar trends are observed between female CHD cases and female healthy controls. In addition, meta-analysis of 8 prospective studies among 77,400 Caucasians provides evidence that 719Arg increases the risk of CHD (P < 0.001, HR = 1.27 95% CI = 1.15-1.41). However, a negative result is observed in another meta-analysis of 7 case-control studies among 65,200 individuals including Europeans or European descendents, African descendents in America, and Asians (P = 0.642, OR = 1.02, 95% CI = 0.95-1.08). This suggests that the contribution of Trp719Arg to the risk of CHD varies in different ethnic groups. A third meta-analysis of 8 studies among 52,000 individuals shows that statin therapy only benefit the vascular patients with 719Arg allele (P < 0.001, relative ratio (RR) = 0.60, 95% CI = 0.54-0.67).  Conclusions: Our result suggests that Trp719Arg of KIF6 gene is associated with the risk of CHD in female Han Chinese. Meta-analysis has established its contribution to the risk of CHD in Caucasians. However, the effects of Trp719Arg on CHD may vary in other ethnic populations. This variant may be used to predict the efficacy of statin therapy in vascular diseases. | 2 |
| **INTRODUCTION** | | |  |
| Rationale | 3 | Kinesin like protein 6 (KIF6) gene encodes an intracellular motor protein transporting cellular cargos along microtubules in an ATP dependent process. KIF6 gene is expressed in many tissues and cell types including coronary arteries and vascular cells. Single nucleotide polymorphisms (SNPs) of KIF6 such as Trp719Arg (rs20455) have been shown to be associated with the risk of CHD. Eight GWAS have demonstrated that 719Arg can increase the risk of CHD in Europeans and North Americans. Moreover, 719Arg carriers may have better statin therapeutic effects that includes the effect of reducing the low-density lipoprotein cholesterol (LDL-C) levels, and other pleiotropic effects on inflammation, thrombogenesis, and arterial vasomotor function. Despite the above evidence, negative association results are observed in different populations. These discrepancies suggest that the role of Trp719Arg in the risk of CHD may vary for different ethnic groups. | 3 |
| Objectives | 4 | The goals of our study are to examine the association between Trp719Arg and the risk of CHD in the Eastern Han Chinese, and to summarize the contribution of Trp719Arg to the risk of CHD and the therapeutic effect of statins. | 3 |
| **METHODS** | | |  |
| Protocol and registration | 5 | No |  |
| Eligibility criteria | 6 | The included studies have to satisfy the following criteria: 1) they have been published as articles or letters in peer-reviewed journals, 2) had a case-control design or a nested case-control design within a prospective study and reported their results by genotype, or had sufficient published data on ORs or HRs and 95% CIs, or genotype and allele frequencies to determine an measure of relative risk. | 5 |
| Information sources | 7 | We systematically search for available articles in English or Chinese from 2005 to 2011 in multiple electronic databases, including PubMed, EMbase, China National Knowledge Infrastructure (CNKI), Wanfang Chinese Periodical Database and Web of Science. | 5 |
| Search | 8 | The search keywords apply the MeSH (Medical Subject Headings in the US National Library of Medicine) terms that include “coronary heart disease” or “coronary artery disease” or “myocardial infarction” combined with “KIF6” or “kinesin like protein 6” or “rs20455” or “719Arg”, “polymorphism” and “genetic association”. We read the full text articles to collect the relevant information. The related articles in the MEDLINE option as well as reference lists of all retrieved studies are also checked for citations of other relevant publications that are not identified initially. | 5 |
| Study selection | 9 | A total of 14 published articles are eligible for the meta-analysis of Trp719Arg to the risk of CHD. Among these studies, 8 are prospective studies with 77,400 individuals (Caucasians in Europe and North America), while the other 6 studies are case-control studies with 65,200 individuals (Europeans or European descendents, North Americans and Asians). In addition, 8 association studies between KIF6 Trp719Arg and statin response are assessed in a third meta-analysis | 5 |
| Data collection process | 10 | Data extraction is carried out by at least two reviewers (PP and LMX) on a standard protocol, and the consensus data are established by discussion. | 5 |
| Data items | 11 | In the meta-analyses, the following data collection is included: name of the first author, publication year, country, ethnic population, study stage, numbers of individual in the case and the control groups and prospective studies, OR, RR, HR and 95% CI. | 5 |
| Risk of bias in individual studies | 12 | Heterogeneity was tested by Cochran’s Q statistic test and I^2^ test. Because Q statistic is vulnerable to the influence of sample size, I^2^ and H statistics were used to evaluate heterogeneity. When the P value <0.05 for the Q test which indicated heterogeneity among the studies, study-specific ORs were pooled by using the random-effects model . Otherwise we used a fixed-effects model. | 6 |
| Summary measures | 13 | OR values of genotypes are determined by comparing the heterozygous or homozygous genotype to wild type. The 95% CI are calculated using the SPSS statistical software (version 18) | 6 |
| Synthesis of results | 14 | The meta-analyses are performed by REVMAN software (version 5.0, Cochrane Collaboration, Oxford, United Kingdom) and Stata software (version 11.0, Stata Corporation, College Station, TX). Publication bias is visualized by funnel plots and Egger regression plot. A two-tailed value of P < 0.05 is considered to be significant. | 6 |

Page 1 of 2

| **Section/topic** | **#** | **Checklist item** | **Reported on page #** |
| --- | --- | --- | --- |
| Risk of bias across studies | 15 | Publication bias was visualized by the funnel plot and the Egger test for asymmetry. | 5 |
| Additional analyses | 16 | We performed two separate meta-analyses for perspective studies and case-control studies between KIF6 Trp719Arg and the risk of CHD. In addition, a third meta-analysis was done betweenTrp719Arg and statin effect. | 7-8 |
| **RESULTS** | | |  |
| Study selection | 17 | A total of 8 prospective studies among 77,400 individuals were included for the first meta-analysis. The other 6 studies plus our case-control studies among 65,200 individuals were included for the second meta-analysis. In addition, eight association studies between KIF6 Trp719Arg and statin response are assessed in a third meta-analysis | 7-8 |
| Study characteristics | 18 | The information extracted from each report included name of the first author, publication year, country, ethnic population, study stage, numbers of individual in the case and the control groups and prospective studies, OR, RR, HR and 95% CI. | 7-8 |
| Risk of bias within studies | 19 | No publication bias is observed among the involved studies in all the three meta-analyses | 8 |
| Results of individual studies | 20 | As shown in Figure 1, we found a significant contribution of 719Arg allele to the risk of CHD (P < 0.001, the overall HR =1.27, 95% CI = 1.15 - 1.41) and a high heterogeneity among the 8 prospective studies (I2 = 64.4%, P = 0.002, χ2 = 28.11, df = 10). In contrast, a low heterogeneity is observed in the meta-analysis of the six case-control studies and our study (I^2^ = 18.6%, χ2 = 15.98, df = 13, P = 0.25). It is unable to find a significant association between 719Arg and the risk of CHD (P = 0.642, the overall OR = 1.02, 95% CI = 0.95-1.08). This meta-analysis is involved with 65,200 individuals from Europeans or European descendents, African descendents in America, and Asians. This suggests that the contribution of Trp719Arg to the risk of CHD varies in different ethnic groups. As shown in Figure 3, eight association studies between KIF6 Trp719Arg and statin response are assessed in the meta-analysis. The meta-analysis shows a significant reduction of the number of deaths or major cardiovascular events in the 719Arg carriers (P < 0.001, overall RR = 0.60, 95% CI = 0.54 - 0.67). High heterogeneity (I2 = 56.5%, P = 0.011, χ2 =23, df = 10) is found among the eight studies. Random effects analysis model is used for the meta-analysis. | 7-8 |
| Synthesis of results | 21 | Random effects analysis model was used for the meta-analyses of prospective studies and the stain response studies. For the meta-analysis of the case-control studies, fix-effect model was used. | 7-8 |
| Risk of bias across studies | 22 | No publication bias is observed among the involved studies in all the three meta-analyses in the figure 2. | 7-8 |
| Additional analysis | 23 | No. |  |
| **DISCUSSION** | | |  |
| Summary of evidence | 24 | Several lines of evidence have shown that 719Arg is likely to increase the risk of CHD. In the Cardiovascular Health Study (CHS), a population-based investigation of 3,849 white Americans found that 719Arg was associated with the risk of cardiovascular disease. Two prospective trials comprising the Cholesterol and Recurrent Events (CARE) and the West of Scotland Coronary Prevention Study (WOSCOPS) have revealed 719Arg as a CHD risk factor among a total of over 4,000 Caucasian participants. Another investigation among 25,283 initially healthy Caucasian women, namely Women's Health Study (WHS), found that females with 719Arg allele of KIF6 have 34% higher risk of AMI and 24% higher risk of CHD. Under a dominant model we reveal the contribution of 719Arg to the higher risk of CHD in females (P = 0.04, χ2 = 4.231, df = 1, OR = 2.015, 95% CI = 1.024 - 3.964). This female-specific finding agrees with the observations in a total of 25,283 Caucasian women enrolled in the WHS. Meta-analyses of 8 studies among 77,400 Caucasians provides evidence that 719Arg increases the risk of CHD (P < 0.001, HR = 1.27, 95% CI = 1.15-1.41). This result agrees with a previous meta-analysis that has found a 20% increase in the risk of CHD for the 719Arg carriers.  We also notice that there is an ethnic difference in the frequency of 719Arg allele. In our healthy controls, it is 0.483 that is similar to 0.570 in HapMap-CHB, 0.477 in HapMap-JPT, and 0.51 in the Indian population. However, much lower frequency of 719Arg allele was observed in Europeans (0.358 in HapMap-CEU) and Japanese (0.386). Interestingly, the latter is much lower than 0.477 in HapMap-JPT that consists of 90 Japanese individuals. The Costa-Rican population, an admixture of three populations, Southern Europeans, Amerindians, and West Africans, has a minor allele frequency of 0.345. It is interesting to find that the 719Arg allele frequency is extremely high in the Sub-Saharan African population (0.908). These ethnic differences imply that further replication of 719Arg to the risk of CHD in other populations is warranted.  Negative association between 719Arg and the risk of CHD is found in the meta-analysis of 7 case-control studies among 65,200 individuals (P = 0.642, OR = 1.02, 95% CI = 0.95-1.08). The recruited participants in the meta-analysis are from several different ethnic populations including Europeans or European descendents, African descendents in America, East Asians, and South Asians. Among these case-control studies, a large one with a total of 17,000 cases and 39,369 controls was unable to replicate the association between Trp719Arg and the risk of clinical CHD in multiple ethnic populations. The contribution of 719Arg to the risk of CHD was unable to be replicated in the Costa Rican and the Western Indian. The conflicting results may be explained by the survival bias and drug interaction that can attenuate the case-control comparisons of Trp719Arg, or it could be also due to the lack of genetic effect in certain ethnic groups.  An allele-specific model of 719Arg is observed in the statin therapy of coronary events. Significantly reduced coronary events and other major vascular events were observed in 719Arg carriers but not in non-carriers, although a large primary prevention trial JUPITER study with 8,781 Caucasian trial participants found no difference in the rosuvastatin therapeutic outcomes between carriers (P = 0.007, HR = 0.61, 95% CI = 0.43 - 0.87) and non-carriers (P = 0.009, HR = 0.59, 95% CI = 0.39 - 0.88) of 719Arg. We summarized the eight studies and found that statin therapy received significant benefit only in the carriers of 719Arg (P < 0.001, overall RR = 0.60, 95% CI = 0.54 - 0.67). | 9-11 |
| Limitations | 25 | There were several limitations in our study. Firstly, sample size in our study was comparatively small and it has only 20.6% power to detect the association of Trp719Arg with CHD at a significant level of 0.05. In addition, only non-fatal CHD cases were recruited in the present study. The 719Arg allele is hypothesized to increase the risk of incident fatal CHD more than the risk of incident nonfatal CHD [34], the exclusion of fatal CHD cases could attenuate the detection of a significant association between the SNP and CHD. Secondly, the genotype distribution of Trp719Arg in female CHD patients has an excess of heterozygotes (HWE test: P = 0.041). This phenomenon may be due to the improperly pair-wised design, the small population size or that the female CHD patients did not obey HWE. Therefore, we need to take caution with the significant association results in females under the dominant model. Thirdly, the information of statin usage was not available in our samples. Our study might underestimate the risk of this mutation for a potential bias by the statin therapy. Moreover, CHD is strongly correlated with smoking status, LDL-C/HDL-C, history of hypertension or diabetes, and body mass index. Our genetic testing did not adjust with those risk factors. Finally, our study wasn’t designed to test whether KIF6 variants are associated with the statin therapy outcome. | 11 |
| Conclusions | 26 | In conclusion, we have identified a female-specific association between Trp719Arg and CHD in the Eastern Han Chinese. Meta-analyses of 15 studies over 143,000 individuals have shown that 719Arg is a risk factor of CHD in Caucasians but its effects on CHD may vary in other ethnic populations. Another meta-analysis of 8 studies among 52,000 individuals has indicated that statin therapy may selectively benefit patients with KIF6 719Arg allele. | 11 |
| **FUNDING** | | |  |
| Funding | 27 | The research was supported by the grants from: National Natural Science Foundation of China (31100919 and 30772155), Qianjiang Scholars Foundation of Zhejiang Province, Zhejiang Provincial Program for the Cultivation of High level Innovative Health Talents, Natural Science Foundation of Zhejiang Province (Y206608), the Scientific Innovation Team Project of Ningbo (No.2011B82014), Youth and Doctor Foundation of Ningbo (2005A610016), Zhejiang Provincial Natural Science Foundation (No.Y2100240), and Ningbo Natural Science Foundation (No.2009A610142). The authors gratefully acknowledge the support of K.C. Wong Education Foundation, Hong Kong. | 12 |

*From:*  Moher D, Liberati A, Tetzlaff J, Altman DG, The PRISMA Group (2009). Preferred Reporting Items for Systematic Reviews and Meta-Analyses: The PRISMA Statement. PLoS Med 6(6): e1000097. doi:10.1371/journal.pmed1000097

For more information, visit: **www.prisma-statement.org**.

Page 2 of 2
